# Hydrogel Polymer Electrolytes: Synthesis, Physicochemical Characterization and Application in Electrochemical Capacitors

**DOI:** 10.3390/gels9070527

**Published:** 2023-06-28

**Authors:** Piotr Gajewski, Wiktoria Żyła, Klaudia Kazimierczak, Agnieszka Marcinkowska

**Affiliations:** Institute of Chemical Technology and Engineering, Poznan University of Technology, Berdychowo 4, 60-965 Poznan, Poland; wiktoria.zyla@student.put.poznan.pl (W.Ż.); klaudia.kazimierczak@student.put.poznan.pl (K.K.); agnieszka.marcinkowska@put.poznan.pl (A.M.)

**Keywords:** hydrogel polymer electrolyte, photopolymerization, electrochemical capacitor

## Abstract

Electrochemical capacitors operating in an aqueous electrolyte solution have become ever-more popular in recent years, mainly because they are cheap and ecofriendly. Additionally, aqueous electrolytes have a higher ionic conductivity than organic electrolytes and ionic liquids. These materials can exist in the form of a liquid or a solid (hydrogel). The latter form is a very promising alternative to liquid electrolytes because it is solid, which prevents electrolyte leakage. In our work, hydrogel polymer electrolytes (HPEs) were obtained via photopolymerization of a mixture of acrylic oligomer Exothane 108 with methacrylic acid (MAA) in ethanol, which was later replaced by electrolytes (1 M Na_2_SO_4_). Through the conducted research, the effects of the monomers ratio and the organic solvent concentration (ethanol) on the mechanical properties (tensile test), electrolyte sorption, and ionic conductivity were examined. Finally, hydrogel polymer electrolytes with high ionic conductivity (σ = 26.5 mS∙cm^−1^) and sufficient mechanical stability (σ_max_ = 0.25 MPa, ε_max_ = 20%) were tested using an AC/AC electrochemical double layer capacitor (EDLC). The electrochemical properties of the devices were investigated via cyclic voltammetry, galvanostatic charge/discharge, and impedance spectroscopy. The obtained results show the application potential of the obtained HPE in EDLC.

## 1. Introduction

The rapid progress of civilization, which resulted in the electronics industry’s rapid development and the need to make devices more compact, led to the requirement for new solutions in the field of energy storage devices [1,2,3]. Recently, electrochemical systems for energy storage devices emerged as the best and most adaptable options for matching power delivery to demand [4]. Around the world, industry and academic experts have become increasingly interested in the development of fresh approaches for energy storage technologies [5]. Electronic systems and goods have become indispensable components of modern life since the development of the transistor in the late 1940s [4].

Given the need for electronic gadgets, such as smartphones and tablets, to maintain their charge and function throughout the day, high-performance energy-storage systems are required to live our daily lives in a connected environment. As was predicted in reviews conducted a few years ago, electrochemical capacitors (ECs) have become ever-more important to meeting the demand for high-rate electrical energy harvesting, storage, and delivery technologies. This demand explains why those energy storage technologies have been given special attention by experts [6].

Electrochemical capacitors (ECs), which can be also found in the literature under the name supercapacitors, are energy storage technologies that store energy via ion adsorption (electrochemical double layer capacitors) or fast surface redox reactions (pseudo-capacitors). Recent improvements in the comprehension of charge storage mechanisms and the development of novel materials and chemicals employed in their fabrication resulted in a considerable improvement in their performance [7,8,9].

The typical electrochemical capacitor in construction comprises two electrodes and an electrolyte, which plays a major role in electrochemical properties [8,10]. The electrolyte is both an ion conductor and a source of positive and negative ions, which explains why it has a huge influence on the performance of ECs. It can have both direct and indirect effects on the power density, capacitance, energy density, thermal stability, cycle life, and equivalent series resistance (ESR) of a supercapacitor [11]. The ideal electrolyte should fulfill the following electrochemical criteria: high ionic conductivity, high chemical and thermal stability, and chemical inertness to cell components. However, it must also be safe, non-toxic, and economically affordable [12].

Electrolytes can be divided into several groups based on their structure, preparation, and electrochemical properties. For safety and environmental protection reasons, water-based electrolytes, which are found in both liquid and solid form (hydrogels), are the most frequently used type. They are often used in research due to their low price, easy availability, and minimal ecological impact [2,13,14]. Furthermore, aqueous electrolytes show other benefits, like lower viscosity and high conductivity values (at least one order of magnitude higher), compared to organic electrolytes. Their toxicity is frequently small; thus, they are environmentally friendly and non-flammable [15].

As mentioned previously, aqueous electrolytes can be found in liquid or solid (hydrogel) form. Hydrogel polymer electrolytes (HPE) have attracted a lot of interest over the past 50 years as a result of their outstanding characteristics and versatility in use. They serve as a separator to prevent direct contact between the electrodes and short circuits, while, as an electrolyte, they enable quick transfer of ions during charging and discharging [16,17]. HPEs ensure no leakage of any liquid component, low vapor pressure, dimensional stability, and reliability [10]. Also, in comparison to organic electrolytes, the cost of production is significantly lower. However, in order to use them in supercapacitors, those electrolytes must simultaneously exhibit high ion conductivity, dimensional stability, and high mechanical strength.

Aqueous electrolytes were mainly developed with either a basic or acidic electrolyte or with neutral aqueous solutions that contain KCl, NaCl, or LiCl salt. Application of these electrolytes highly reduces the maximum cell potential to 0.8–1 V (the evolution of hydrogen, oxygen, or chloride above 1 V) [18,19,20,21]. The potential of ECs can be extended up to 1.6 V by applying neutral electrolytes composed of sulfate or nitrate salts [2,22,23,24].

HPE can be synthesized via sorption of an aqueous electrolyte into a polymer matrix obtained using commercially available polymers, such as polyvinylpyrrolidone (PVP), potassium polyacrylate (PAAK) [11], poly(vinyl alcohol) (PVA) [12,13,18], and others [14,15,16,17,25]. However, this method highly limits modification of the HPE structure. Therefore, polymerization of selected monomers seems to be the better option, because it allows for precise design of the final properties of synthesized HPEs. In particular, the use of photopolymerization to initiate the polymerization process seems to be the best method to obtain HPE. Photopolymerization has numerous advantageous features, such as excellent spatial and temporal controllability, as well as the fact that reaction occurs in room temperature without additional heating, meaning that energy demand is very low and the whole procedure is economical. This procedure only takes a few minutes, and there are no byproducts [20,21,26,27,28].

Currently, several directions of research on hydrogel polymer electrolytes can be distinguished. These directions include, among others, the search for a new polymer matrices [25,29,30,31], the use of electrolytes with various salts and their mixtures [25,31], and the use of electrode materials or electrolytes containing redox compounds to obtain higher capacities (pseudocapacitors) [24,32,33]. Also, recent literature reports on HPEs containing Li_2_SO_4_, Na_2_SO_4_, or LiNO_3_ can be found [22,23,24,26]. These salts are a very common choice for use with neutral liquid and gel electrolytes because their solutions are characterized by high conductivity and, at the same time, they have a much wider electrochemical stability window compared to chlorides.

In the present work, we obtained HPE, which was characterized by high ionic conductivity and had appropriate mechanical properties, which enabled the use of HPE in an electrochemical capacitor. For this purpose, the synthesis of polymer matrix was carried out via the photopolymerization of Exothane 108 oligomer with methacrylic acid in ethanol. After polymerization, the 1 M Na_2_SO_4_ was used as an aqueous phase to conduct EtOH exchange to obtain HPEs. Comparing the HPE obtained by our team and used in EDLC with other HPEs presented in the literature, our hydrogels are shown to have one of the highest conductivities relative to HPEs containing different neutral salts and discussed in recent literature reports, i.e., Li_2_SO_4_ [23], Na_2_SO_4_ [22,24], or LiNO_3_ [26]. In addition, the presented HPE was investigated mechanically, and the results obtained were comparable to or better than those obtained by other authors [23,25]. It should also be noted that mechanical investigations of HPE are very rarely presented in the literature’s reports. Obtaining such good results was probably possible thanks to the use of a properly selected composition of monomers and their subsequent polymerization (selection of the appropriate composition allows us to obtain polymer matrices with very different properties, as shown in the manuscript). This method is superior to the application of ready-made polymers which is the most common method used in the literature, but which does not allow for such extensive changes in the properties of the obtained HPE.

## 2. Results and Discussion

### 2.1. FTIR–ATR Spectra Analysis

**Figure 1:**
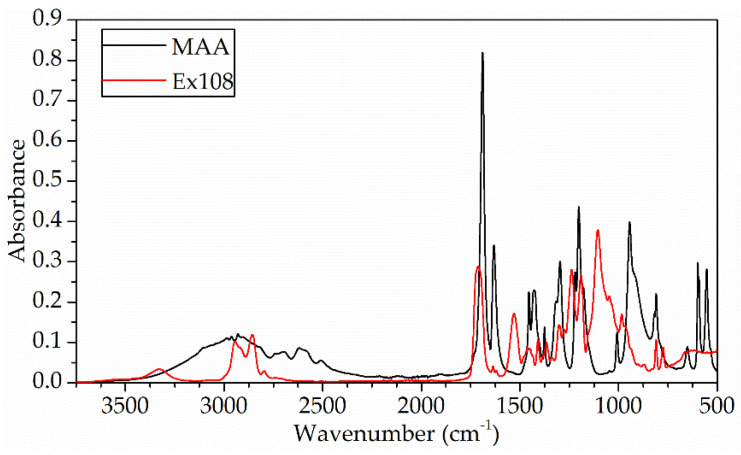
FTIR–ATR spectra of methacrylic acid (MAA) and oligomer Ex108.

In Figure 1, FTIR–ATR spectra of methacrylic acid (MAA) and Exothane 108 (Ex108) are presented. For the spectrum of methacrylic acid, absorption bands of characteristic functional groups, i.e., methacrylic and carboxylic, can be seen. The absorption band at the wavenumber 1632 cm^−1^ corresponds to the stretching vibrations in the C=C double bond. In addition, the vibration in the pendant vinyl group of methacrylate is observed at 810 cm^−1^. At a wavenumber of 1689 cm^−1^, a complex, broad band of vibrations in the carbonyl group derived from the carboxylic acid and the methacrylic group appears. The stretching band of the hydroxyl group of the carboxylic acid is wide and occurs in the range of 3300–3000 cm^−1^. At approximately 2930 cm^−1^, there is a band of stretching vibrations in the CH_2_ group. In contrast, in the spectrum of Exothane, 108 bands related to the presence of urethane and acrylic groups can be seen. Absorption bands originating from the urethane group are the stretching band derived from N-H bonds at wavenumber 3327 cm^−1^, while at the deformation band at a wavenumber of 1530 cm^−1^, the vibration band in the C-N bond is observed at a wavenumber of 1237 cm^−1^. The band of the carbonyl group C=O, as can be seen, is wide and occurs in the range of 1750–1670 cm^−1^, which is related to the presence of this group in both urethane and acrylate, as well as being associated with the formation of hydrogen bonds. The unsaturated bond of the C=C group of the acrylic group occurs at a wavenumber of 1619 cm^−1^ and 1636 cm^−1^, as well as at the C-O-C of the ester of 1105 cm^−1^. Similar to methacrylic acid, the vibration in the pendant vinyl group of acrylate is observed at 810 cm^−1^. The stretching vibrations in the CH_2_ group occur at a wavenumber of 2944 cm^−1^.

**Figure 2:**
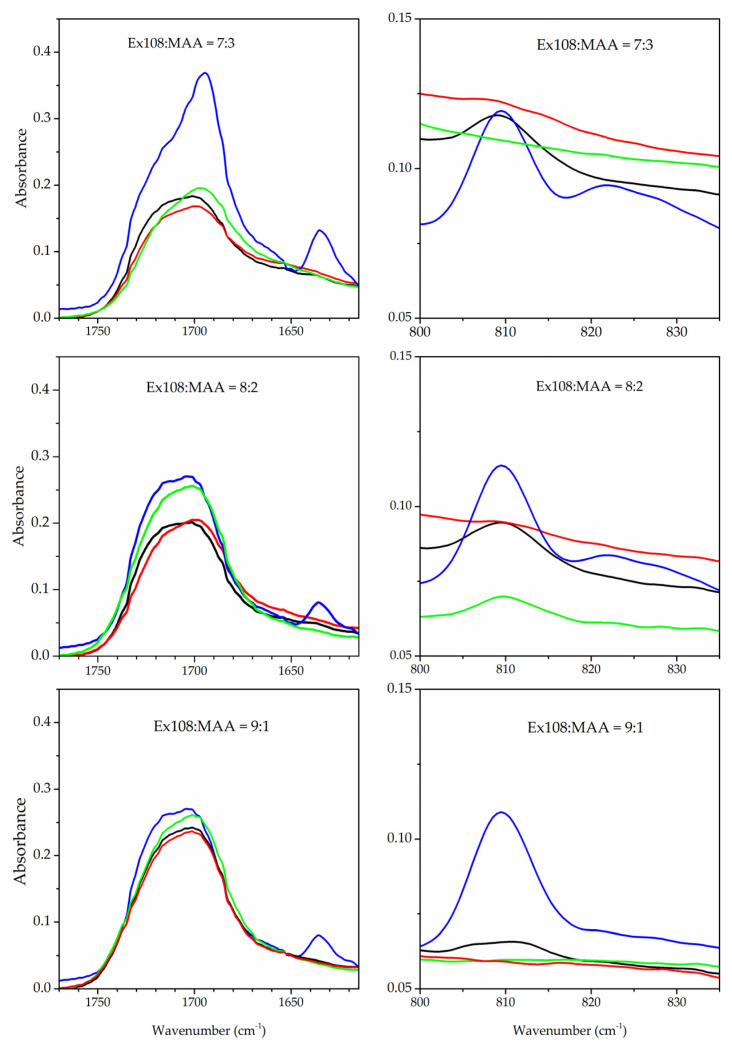
FTIR–ATR spectra of the photocurable composition before polymerization (**―**, composition without the solvent) and with the polymer matrix with different amounts of solvent during the polymerization step: **―** 70%; **―** 60%; **―** 50% of solvent; the solvent was evaporated from polymer matrix before FTIR-ATR measurement.

FTIR–ATR spectra were recorded for all tested compositions before and after photopolymerization to assess whether the (meth)acrylic groups reacted during the reaction. For this purpose, the heights of the absorption bands of the unsaturated C=C bond originating from both methacrylic acid and Ex108 were studied. Figure 2 shows the spectra in the range of occurrence in these vibrations, which are around 1630 cm^−1^ (together with bands of the C=O group) and 810 cm^−1^. As can be seen, the (meth)acrylic groups did not react at 100%, and the spectra absorption bands originating from the unsaturated C=C bond can be seen. The degree of conversion increases as the Ex108 content in the composition changes from 7:3 to 9:1. In addition, a higher concentration of the solvent causes a lower conversion (higher absorption of the bands) of the (meth)acrylic groups. Thus, the highest conversion is observed for composition Ex108:MAA 9:1 in 50% of the solvent, and the lowest conversion for composition Ex108:MAA 7:3 is observed in 70% of the solvent. Thus, a network with a higher density is obtained in the case of systems containing a higher concentration of Ex108 and a lower concentration of the solvent. This result may be related to the longer chain length of the oligomers, which means that they are able to react with each other in a dilute environment. The obtained results are related to the mechanical properties of the tested hydrogels.

### 2.2. Electrolyte Sorption

**Figure 3:**
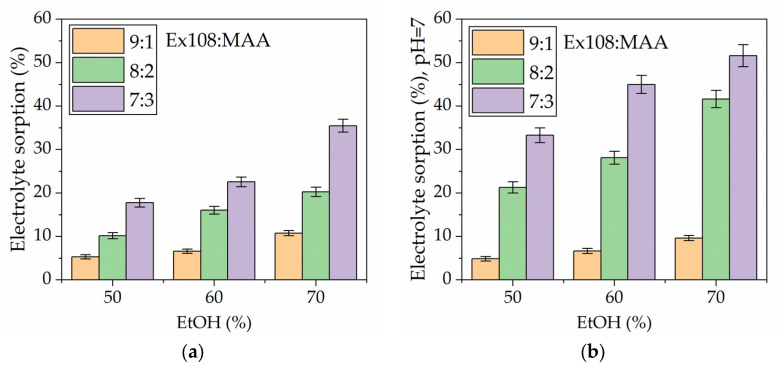
The sorption of 1 M Na_2_SO_4_ electrolyte (**a**) without or (**b**) with neutralization to roughly pH = 7.

According to the described procedure (Section 4.2.1), the synthesis was carried out via solvent polymerization in EtOH. The resulting gel was then placed in water to wash the solvent (EtOH) out of its structure and replace it with water. Next, the obtained hydrogel was immersed in the electrolyte (1 M Na_2_SO_4_) and, after reaching equilibrium sorption, the weights of the samples were checked. Next, the electrolyte was neutralized to roughly pH = 7, and the weight of the samples was once again measured. As can be seen in Figure 3, the composition of the polymerization mixture has a significant influence on the electrolyte sorption, both before and after neutralization. The hydrogels with the highest weight ratio of Ex108 to MAA (9:1) present the lowest sorption of the electrolyte. Thus, increasing the EtOH content during the polymerization step causes an increase in the sorption from 5 to 10%, though no significant difference in the sorption of hydrogels before and after neutralization is observed. Such low sorption values are the results of high content of the hydrophobic and crosslinking Ex108 oligomer present in the hydrogel. Therefore, the hydrogel structure is not able to swell. Also, the neutralization of MAA in the hydrogel does not cause the sorption to increase, mainly because the amount of MAA in the polymer structure is small, while the additional physical crosslinking performed by hydrogen bonds caused by this monomer is negligible in comparison to the crosslinking caused by Ex108. Therefore, neutralization of the hydrogel does not change the crosslinking densities significantly, and the influence of the neutralization on the sorption is negligible.

In cases of higher content of MAA in the polymer structure, the higher sorption of the electrolyte can be observed. Additionally, after neutralization, even further increasing of the electrolyte sorption can be seen. This phenomena can be explained based on increasing content of MAA in the hydrogel’s structure; therefore, decreasing crosslinking densities and increasing the hydrophilicity (based on the presence of carboxilic groups) of the polymer network. This outcome causes water sorption to increase to roughly 10–20% for Ex108:MAA = 8:2 and to roughly 20–35% for Ex108:MAA = 7:3, depending on the amount of EtOH used during the polymerization step. Additionally, neutralization of the electrolyte causes even further sorption, which increases up to roughly 20–40% and 30–50% for Ex108:MAA = 8:2 and Ex108:MAA = 7:3, respectively. These results clearly show that introduction of a hydrophilic monomer significantly influences electrolyte sorption, and because the MAA is an acidic monomer with the ability to cause hydrogen bond formation, the neutralization of the electrolyte allows us to further increase the sorption. Such a change in electrolyte sorption is typical for hydrogels containing acidic groups in their structure. As a result of the neutralization of these groups, the sorption of the electrolyte increases.

### 2.3. Ionic Conductivity

**Figure 4:**
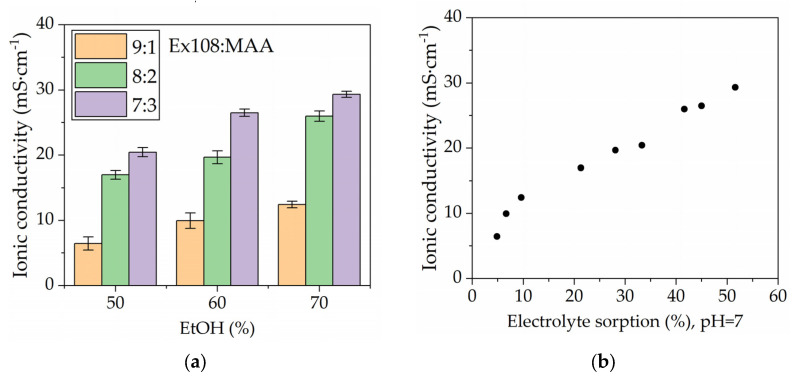
The dependence of ionic conductivity on (**a**) the photocurable mixture composition and (**b**) the electrolyte sorption (after neutralization).

The ionic conductivity measurements were performed according to the methodology described in Section 4.2.5. The analysis of obtained results shows that both the mass fraction of the monomers and the EtOH amount obtained during polymerization had a significant impact on the ionic conductivity (Figure 4a). The ionic conductivity values increased with the increase in the content of the methacrylic acid in the hydrogel (at a constant value of the solvent used). The same relationship was observed when increasing the mass fraction of the solvent in the mixture (at a constant weight ratio of Ex108:MAA). Changing the composition of the photocurable mixture allows us to obtain the HPE with ionic conductivity in the range of roughly 6 mS∙cm^−1^ for the system containing 50% ethanol and with a weight fraction of Ex108:MAA of 9:1 and roughly 30 mS∙cm^−1^ for the system containing 70% ethanol with a weight fraction of Ex108:MAA of 7:3. Taking into account the ionic conductivity of the electrolyte (1 M Na_2_SO_4_), which was 80 mS∙cm^−1^, the relative conductivity values were obtained in the range of roughly 8–37%.

The ionic conductivity is directly correlated with electrolyte sorption (Figure 4b). As expected, the increase in the electrolyte mass fraction after sorption results in an increase in the ionic conductivity of the obtained HPEs. Therefore, the composition of the photocurable mixture affects the obtained sorption values and, thus, the ionic conductivity.

The conductivity values of the HPE obtained in this work shows the conductivity up to 20–30 mS∙cm^−1^, which is a relatively high value compared to the values presented in the literature (HPE containing neutral salts: Li_2_SO_4_ [23], Na_2_SO_4_ [22,24] or LiNO_3_ [26]).

### 2.4. Mechanical Properties

**Figure 5:**
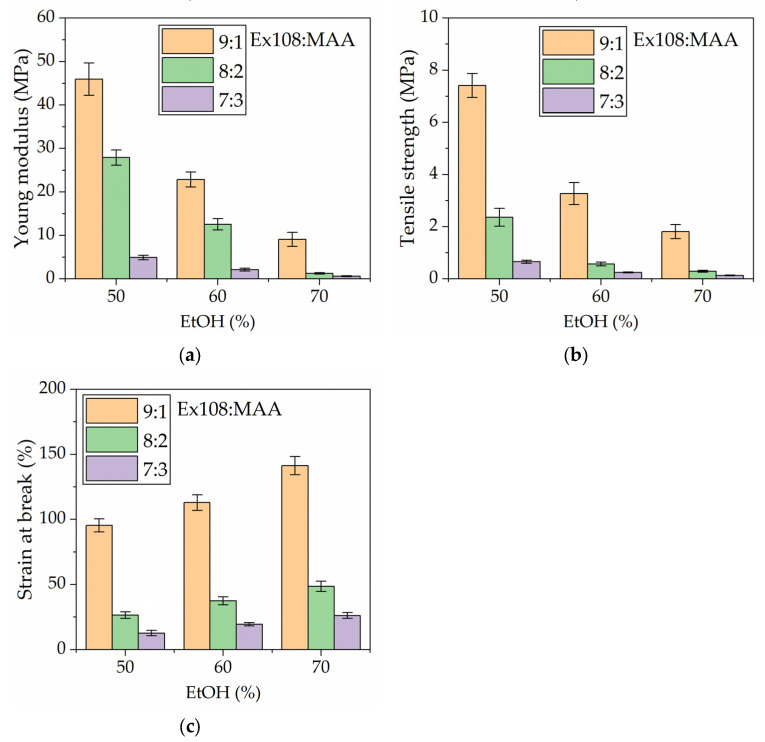
Mechanical properties of the synthesized hydrogel polymer electrolytes. (**a**) Young modulus, (**b**) tensile strength, and (**c**) strain at break. Studies were performed on hydrogels after neutralization.

In Figure 5, the results of tensile tests for hydrogels after neutralization are presented. The obtained values vary over a wide range. The Young’s modulus (Figure 5a) and tensile strength (Figure 5b) values decrease with the decreasing weight ratio of Ex108:MAA (at constant values of the solvent used), as well as while increasing the content of EtOH used for synthesis (at a constant Ex108:MAA weight ratio). For the ratio of Ex108:MAA = 9:1, the Young’s modulus and tensile strength values decreased with increasing EtOH content from roughly 45 MPa to 9 MPa and from roughly 7 MPa to 2 MPa, respectively. For the ratio of Ex108:MAA = 8:2, these values decreased from roughly 28 MPa to 1 MPa and from rough 2 MPa to 0.3 MPa, while for EX:MAA = 7:3, the Young’s modulus and tensile strength values decreased from roughly 5 MPa to 0.5 MPa and from roughly 0.7 MPa to 0.1 MPa, respectively.

At the same time, it is shown in Figure 5c that the strain at break shows the highest values for hydrogels with the highest Ex108:MAA ratio (the range from roughly 95% to 140%), and the lowest values at the lowest Ex108:MAA ratio (the range from roughly 13% to 26%). Similar tendencies were obtained for Young’s modulus and tensile strength. However, the strain at break slightly increases with increasing EtOH content in the photocurable compositions, which is opposite behavior to that evidenced by Young’s modulus and tensile strength.

By comparing the values of the tensile strength and strain recorded at break for HPEs used later in the EDLC with the values presented in the literature, it can be seen that mechanical properties are, in most cases, better for our HPE. For example, Zhao et al. received HPE (PVA with Li_2_SO_4_ solution) characterized by the same stress but had 10 times lower strain [23]. Tan et al. presents HPE (poly(acrylamide-co-isopropylacrylamid) with LiOTf solution), which was characterized by 4–5 times higher strain at break but over 10 times lower tensile strength [25].

### 2.5. Electrochemical Investigation

HPE with the ratio of Ex108:MAA = 7:3 and 60% EtOH content in the photocurable composition was selected for the electrochemical tests. This HPE (thickness roughly 300 μm) was characterized by the high conductivity (σ = 26.5 mS∙cm^−1^) and sufficient mechanical stability (σ_max_ = 0.25 MPa, ε_max_ = 20%), which allowed easy assembling of the capacitor.

**Figure 6:**
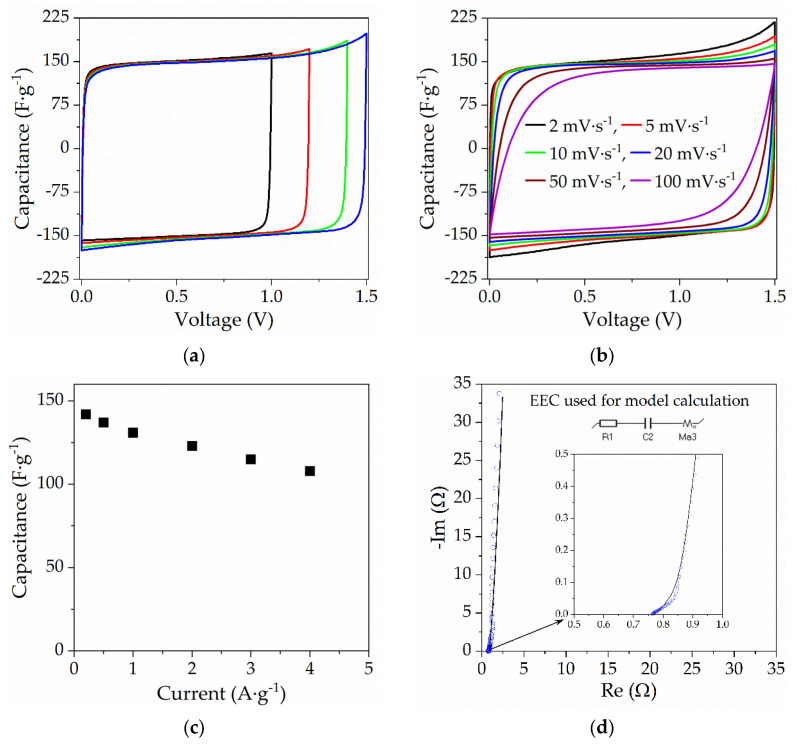
The results of the investigation into the AC/AC electrochemical capacitor with applied HPE. (**a**) Cyclic voltammograms up to various values of maximum potential, with a scan rate 5 mV∙s^−1^; (**b**) cyclic voltammograms at scan rates from 2 to 100 mV∙s^−1^; (**c**) dependence of discharge capacitance on current; and (**d**) Nyquist plot with a fitted model.

The CVs of the AC/AC electrochemical capacitor (Figure 6a) obtained at voltages up to 1.5 V were close to the perfect box-like shape. The absence of peaks on the CV curve proves the capacitive behavior of EDLC without redox reaction. As can be seen in Figure 6b, even increasing the scan rate to 100 mV∙s^−1^ causes only a slight deviation from the ideal box-like shape (maintaining 75% of the capacitance obtained at 2 mV∙s^−1^). In Figure 6c, dependence of the discharge capacitance on current is presented. Current changing from 0.2 A∙g^−1^ to 4 A∙g^−1^ caused a decrease in capacitance from roughly 140 F∙g^−1^ to 110 F∙g^−1^, which corresponds to a decrease of roughly 20% of its initial value.

The Nyquist plot of the electrochemical capacitor (Figure 6d) shows that equivalent series resistance (ESR) of EC with applied HPE is roughly 0.76 Ω (after area normalization is 3.8 Ω∙cm^2^, the resistance of HPE is equal 1.1 Ω∙cm^2^, and the remainder is the resistance of other parts of EC).

As the obtained results show, the EC with HPE is characterized by very good working parameters. There is little decrease in the capacitance with the increasing scan rate or current density. Taking into account the obtained results, the use of HPE is very promising, especially considering that the electrolyte used is non-toxic, and thanks to its solid structure, there is no possibility of its leakage even after damaging the EC.

## 3. Conclusions

The described studies present the results of the influence of HPEs’ composition on their ionic conductivity and mechanical properties. The appropriate selection of the polymer matrix composition allows us to change the mechanical and conductive properties of the synthesized HPEs. The introduction of a hydrophilic, non-crosslinking monomer (MAA) into the structure of the polymer network causes a significant increase in the electrolyte sorption and, thus, an increase in HPEs ionic conductivity. This result is due to the fact that increasing the MAA content causes a decrease in the crosslinking density of the polymer network and an increase in its hydrophilic properties, which results in higher electrolyte sorption and higher HPE ionic conductivity. In contrast, increasing the Ex108 content has the opposite effect on the electrolyte sorption and ionic conductivity, yet, at the same time, it results in an improvement in the mechanical properties of HPEs. Taking into account the application of the obtained hydrogels as HPEs in electrochemical capacitors, it is necessary that they are characterized by high ionic conductivity, while maintaining dimensional stability, thus enabling them to be easily manipulated during the assembly of electrochemical capacitors. As was shown, the application of HPE with high ionic conductivity allowed the assembly and testing of an electrochemical capacitor that gave very high efficiency of EC. The electrochemical capacitor was characterized by high capacitances, even at a high scanning rate and high discharge current. The obtained results show that the appropriate selection of the composition makes it possible to obtain HPE with properties that enable its use in EC. Electrochemical capacitors operating in an aqueous environment are a very promising direction for research and future applications, as they are much more environmental friendly and cheap to manufacture compared to their counterparts that operate in an organic environment.

## 4. Materials and Methods

### 4.1. Materials

Difunctional aliphatic acrylate oligomer Exothane 108 (Ex108) was delivered by ESSTECH (Esstech Inc., Essington, PA, USA), and methacrylic acid (MAA) was delivered by Sigma-Aldrich (St. Louis, MO, USA). The monomers were used without further purification. Ethanol (EtOH), purity ≥ 98%; sodium sulfate (Na_2_SO_4_), purity ≥ 99%; sodium hydroxide (NaOH), purity ≥ 98%; and photoinitiator, 2,2-dimethoxy-2-phenylacetophenone (DMPA) were provided by Sigma-Aldrich (Sigma-Aldrich, St. Louis, MO, USA).

### 4.2. Methodology

#### 4.2.1. Hydrogel Preparation

**Figure 7:**
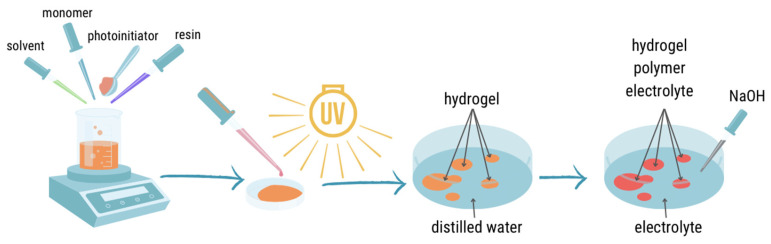
Schematic diagram of HPE preparation.

The hydrogels’ preparation (Figure 7) begin with the preparation of photocurable compositions in the appropriate mass fraction (Table 1) in glass vials. The used compounds were as follows: methacrylic acid, Exothane108 oligomer, photoinitiator-DMPA (0.2 wt.% on the whole composition), and solvent ethanol. The vials were secured with parafilm and then placed in a mechanical shaker until all mixtures became homogeneous solutions. Next, the obtained solutions were poured into a glass molds with thicknesses of 0.3 mm and exposed to UV light for 10 min on each side of the mold (ASN-36W UV lamp, light intensity of 6 mW∙cm^−2^) at room temperature. After photopolymerization, the samples were transferred to containers with distilled water to exchange EtOH for H_2_O and obtain hydrogels. After about 24 h, the obtained hydrogels were transferred from water to water electrolyte – 1 M sodium sulphate (VI)-Na_2_SO_4_ for the next 24 h. Furthermore, the hydorgels were neutralized using a 1 M sodium hydroxide solution to obtain a pH of 6.9–7.0. After this stage, when the electrolyte sorption equilibrium was reached, the hydrogel polymer electrolytes (HPEs) were obtained and used for proper tests.

#### 4.2.2. FTIR-ATR Analysis

Infrared spectra were performed via a Nexus Nicolet 5700 Fourier Transform Infrared Spectrophotometer (FTIR, Thermo Electron Scientific Instruments Corporation, Madison, WI, USA) equipped with an attenuated total reflection (ATR) accessory with a diamond crystal at room temperature in the range 4000–600 cm^−1^ and resolution 4 cm^−1^ at 64 scans. The spectra were collected for photocurable compositions without the addition of a solvent, and polymer matrices were determined after removing water from hydrogels (vacuum dryer: temperature 60 °C and time 12 h, using a vacuum desiccator over a drying agent).

#### 4.2.3. Electrolyte Sorption

Electrolyte sorption was carried out for samples of hydrogels after reaching equilibrium electrolyte sorption, both before and after the neutralization process. The samples were gently dried of excess liquid on the surface, and their mass was weighed. They were then dried in an oven overnight and weighing was repeated. Taking into account the weight of the sample before and after drying, the mass fraction of electrolyte (X_wt,el_) and electrolyte after neutralization (X_wt,neutr_) in the HPE could be calculated using Equation (1).

X_wt,el_ or X_wt,neutr_ = (m_gel_ − m_dry_)/m_gel_, (1)

where X_wt,el_ and X_wt,neutr_ are the mass fractions of the electrolyte before and after neutralization, respectively; m_gel_ is the weight of HPE; and m_dry_ is the mass of dry polymer.

#### 4.2.4. Mechanical Properties

The tensile test was performed on the samples using a CT3 texture analyzer (Ametek Brookfield, Middleboro, MA, USA) after the electrolyte neutralization stage. To determine the cross-sectional area, the thicknesses and widths of undamaged hydrogels were measured. Next, samples were fixed in the clamps of the heads and investigated. The tests were carried out until the destruction of the samples, and the dependences of stress (σ) and strain (ε) were recorded (tensile rate—0.3 mm∙s^−1^, initial force—0.02 N, strain range for Young’s modulus determination—0 to 1%). Measurements were carried out on a minimum of five samples for each HPE composition. On the basis of the obtained results, the tensile strength (σ_max_), Young’s modulus (E_mod_), and strain at break (ε_max_) were determined.

#### 4.2.5. Ionic Conductivity

The measurement of the ionic conductivity for obtained samples was carried out via electrochemical impedance spectroscopy using SP-300 potentiostat/galvanostat (Biologic, Seyssinet-Pariset, France). The study was carried out at room temperature using a two-electrode (stainless 316 L) electrochemical vessel. Samples of hydrogels used for testing were prepared in the shape of circles with diameters of 13 mm. The measurement was carried out for two samples from each HPE composition, and each sample was twice tested. The ionic conductivity of the HPE (σ) was calculated via Equation (2):

σ = σ_s_ ∙ L/A, (2)

where σ is the ionic conductivity of HPEs, mS·cm^−1^; L is the HPE thickness, cm; A is the HPE surface area, cm^2^; and σ_S_ represents the volumetric conductance, mS.

#### 4.2.6. Electrochemical Investigation

##### Preparation of Electrodes and Electrochemical Capacitor

The carbon electrodes were prepared by mixing the appropriate amount of activated carbon—85 wt.% of Maxsorb MSP-20X (Kansai Coke and Chemi-calsco) with carbon black, 5 wt.% of C65 (Imerys) and binder, 1.5 wt.% carboxymethyl cellulose, 0.5 wt.% Laponite-RD (BYK-Chemie GmbH, Abelstrasse 45, Wesel, Germany), and 8 wt.% LITEX LB-420 (Synthomer (UK) Limited, Central Road, Templefields Harlow, United Kingdom). The suspension of electrode material was prepared by mixing of all ingredients in deionized water until a homogeneous suspension was obtained. Next, the thin suspension film was applied to an etched stainless steel (316 L) current collector coated with Acheson electrodag PF-407C (Henkel) using the automatic film applicator Bevs 1811/3 (BEVS Industrial Co., Ltd., Shengli Technology Park No.257, Huangpu District Guangzhou City, China). Next, the water was evaporated, and the electrode sheet was obtained.

##### Electrochemical Capacitor Investigations

The HPE was investigated in a two-electrode symmetric AC/AC pouch-cell-type electrochemical capacitor. The experiments were carried out using an SP-300 potentiostat/galvanostat (Biologic, France). Before assembling the pouchcell, the 20 mm × 25 mm carbon electrodes were soaked off in a 1 M Na_2_SO_4_, and the 25 mm × 30 mm HPE was used as a separator and electrolyte. The average mass of activated carbon in the electrode was roughly 25 mg. Later on, the AC mass in one electrode was used for current density and specific capacitance calculation. The cells were investigated via cyclic voltammetry (CV, scan rates from 2 to 100 mV·s^−1^ and up to cell potentials of 1 to 1.5 V), galvanostatic charge/discharge with potential limitation (0.2 to 4.0 A·g^−1^, calculated based on the average AC mass in one electrode), and electrochemical impedance spectroscopy at an open circuit voltage (OCV) (EIS, over the frequency range from 1 MHz to 1 mHz with a 10 mV amplitude).

## Figures and Tables

**Table 1:** Formulations of the investigated compositions (Ex108 and MAA)/ethanol (with 0.2 wt.% of DMPA photoinitiator).

Formulation	Ex108	MAA	EtOH
	wt.%		
Ex108:MAA = 9:1_EtOH50	45	50	5
Ex108:MAA = 8:2_EtOH50	40	50	10
Ex108:MAA = 7:3_EtOH50	35	50	15
Ex108:MAA = 9:1_EtOH60	36	60	4
Ex108:MAA = 8:2_EtOH60	32	60	8
Ex108:MAA = 7:3_EtOH60	28	60	12
Ex108:MAA = 9:1_EtOH70	27	70	3
Ex108:MAA = 8:2_EtOH70	24	70	6
Ex108:MAA = 7:3_EtOH70	21	70	9

## Data Availability

The data presented in this study are available upon request from the corresponding author.
